# Alpine salamanders at risk? The current status of an emerging fungal pathogen

**DOI:** 10.1371/journal.pone.0298591

**Published:** 2024-05-17

**Authors:** Philipp Böning, Stefan Lötters, Benedetta Barzaghi, Marvin Bock, Bobby Bok, Lucio Bonato, Gentile Francesco Ficetola, Florian Glaser, Josline Griese, Markus Grabher, Camille Leroux, Gopikrishna Munimanda, Raoul Manenti, Gerda Ludwig, Doris Preininger, Mark-Oliver Rödel, Sebastian Seibold, Steve Smith, Laura Tiemann, Jürgen Thein, Michael Veith, Amadeus Plewnia

**Affiliations:** 1 Department of Biogeography, Trier University, Trier, Germany; 2 Department of Environmental Science and Policy, University of Milan, Milan, Italy; 3 St. Michael College, Zaandam, Netherlands; 4 Department of Biology, University of Padova, Padova, Italy; 5 National Biodiversity Future Center, Palermo, Italy; 6 Technisches Büro für Biologie, Absam, Austria; 7 Independent Researcher, Zeil am Main, Germany; 8 UMG Umweltbüro Grabher, Dornbirn, Austria; 9 Centre d’Ecologie et des Sciences de la Conservation (CESCO), Muséum National d’Histoire Naturelle, Centre National de la Recherche Scientifique, Sorbonne Université, Paris, France; 10 Auddicé Biodiversité–ZAC du Chevalement, Roost-Warendin, France; 11 Museo Nacional de Ciencias Naturales, CSIC, Madrid, Spain; 12 Konrad Lorenz Institute of Ethology, University of Veterinary Medicine Vienna, Vienna, Austria; 13 Amphibienwerkstatt, Innsbruck, Austria; 14 Vienna Zoo, Vienna, Austria; 15 Museum für Naturkunde–Leibniz Institute for Evolution and Biodiversity Science, Berlin, Germany; 16 Forest Zoology, Technische Universität Dresden, Tharandt, Germany; 17 Berchtesgaden National Park, Berchtesgaden, Germany; 18 Ecosystem Dynamics and Forest Management, Technical University of Munich, Freising, Germany; 19 Department of Neurology, TUM School of Medicine, Technical University of Munich, Munich, Germany; 20 Büro für Faunistik und Umweltbildung, Haßfurt, Germany; Universitat Zurich, SWITZERLAND

## Abstract

Amphibians globally suffer from emerging infectious diseases like chytridiomycosis caused by the continuously spreading chytrid fungi. One is *Batrachochytrium salamandrivorans* (*Bsal*) and its disease ‒ the ‘salamander plague’ ‒ which is lethal to several caudate taxa. Recently introduced into Western Europe, long distance dispersal of *Bsal*, likely through human mediation, has been reported. Herein we study if Alpine salamanders (*Salamandra atra* and *S*. *lanzai*) are yet affected by the salamander plague in the wild. Members of the genus *Salamandra* are highly susceptible to *Bsal* leading to the lethal disease. Moreover, ecological modelling has shown that the Alps and Dinarides, where Alpine salamanders occur, are generally suitable for *Bsal*. We analysed skin swabs of 818 individuals of Alpine salamanders and syntopic amphibians at 40 sites between 2017 to 2022. Further, we compiled those with published data from 319 individuals from 13 sites concluding that *Bsal* infections were not detected. Our results suggest that the salamander plague so far is absent from the geographic ranges of Alpine salamanders. That means that there is still a chance to timely implement surveillance strategies. Among others, we recommend prevention measures, citizen science approaches, and ex situ conservation breeding of endemic salamandrid lineages.

## Introduction

Globally, amphibian declines and extinctions occur due to multiple factors and on a broad taxonomic scale [1, 2]. One of the most important drivers is chytridiomycosis, an emerging infectious disease (EID) induced by parasitic skin fungi that have caused massive amphibian declines and extinctions globally [3]. Among them is *Batrachochytrium salamandrivorans* (*Bsal*) which is a threat to caudate amphibians in the Western Palearctic [3, 4]. It is also referred to as the agent of the ‘salamander plague’ and was detected in Europe at least two decades ago, likely introduced from Asia [5]. So far, outbreaks in wild urodelans have been reported from Belgium, Germany, the Netherlands, and Spain [6–11]. Most of the temperature regimes in Europe appear suitable for *Bsal* and despite active dispersal ability being low, massive range expansions have been observed, which are likely human-mediated and presumably attributed to rapid changes in the pathogen’s thermal optimum [8, 9, 12–14]. Moreover, of the 40 urodelan species in Europe, 30 are considered at high risk of at least local extinction due to *Bsal* until year 2030 [15].

**Fig 1:**
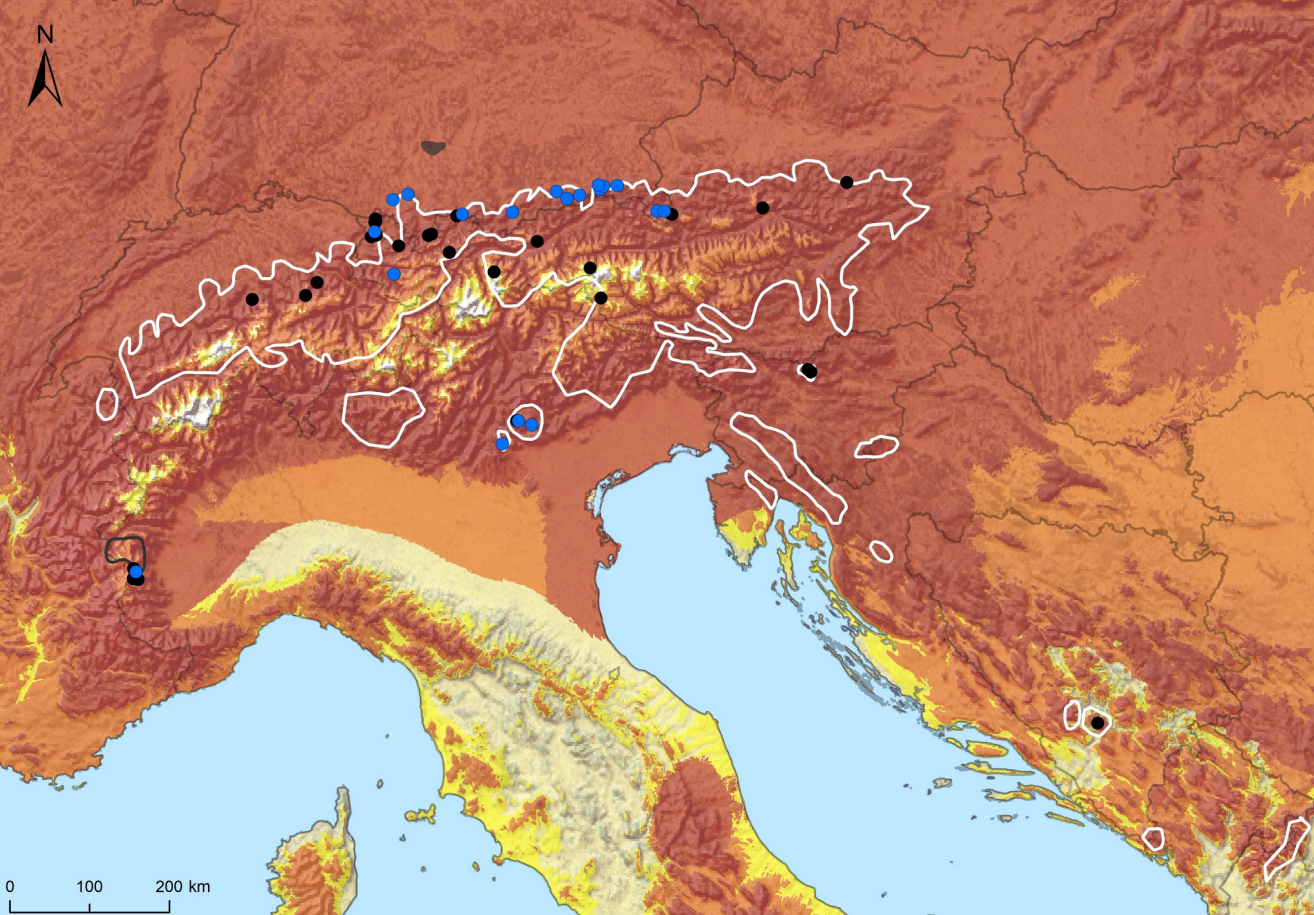
Study sites, distributional ranges of Alpine salamanders and *Bsal* suitability (white polygon line = *S*. *atra*, range adapted from [18, 22]; dark grey polygon line = *S*. *lanzai* adapted from [23], grey polygon = *Bsal* range in Allgovia, Germany); blue points correspond to localities sampled in this study, black points to sampling sites from literature. The yellow highlighted areas refer to MTP estimation, the orange highlighted areas belongs to the MTS estimation and the red highlighted areas belong to the 10^th^TP estimation (see Methods). The map was created by authors in ArcGIS with base maps provided by Eurostat (GISCO, https://ec.europa.eu/eurostat/web/gisco/geodata/reference-data/administrative-units-statistical-units/nuts) and Natural Earth (naturalearthdata.com).

Alpine salamanders (*Salamandra atra* and *S*. *lanzai*) belong to the most imperilled herpetofauna of Europe (Fig 1) [15–17]. They are restricted to the European Alps and the Dinarides and well known for their biology with a viviparous reproductive mode. *Salamandra atra* comprises several intraspecific lineages of which some have been described as subspecies while others remain unnamed [18]. For these subspecies (except *S*. *a*. *atra*) as well as the poorly studied *S*. *a*. *prenjensis* data on distribution and conservation status are widely lacking, hampering a thorough assessment. *Salamandra atra aurorae*, *S*. *a*. *pasubiensis* and *S*. *lanzai* have very small geographic ranges (≤100km^2^) [18, 19] (Fig 1) and are in high risk of total extinction due to further spread of *Bsal* [15]. *Bsal* was recently detected in southern Germany at a straight-line distance of approximately 50 km from known *S*. *a*. *atra* localities [20]. This species is known to be highly susceptible to *Bsal* in captivity [21], which is of great concern for Alpine salamanders.

Despite a Europe-wide call for action against the pathogen [14], no broad *Bsal*-screening throughout the Alps and Dinarides has been carried out so far. Moreover, comprehensive host species monitoring programs are lacking [24–27]. We therefore performed a study delineating the status of the *Bsal*-infection in populations of Alpine salamanders and included data from the Austrian *Bsal* monitoring project established in 2016. The goals were (1) to summarize available data on *Bsal* infections in wild hosts in the Alpine region, (2) to provide a first comprehensive *Bsal*-screening on *S*. *a*. *atra* in the Northern Alps and the local endemics *S*. *lanzai*, *S*. *atra aurorae* and *S*. *a*. *pasubiensis* from the Southern Alps and, (3) to review and enhance pre- and post-exposure mitigation strategies and recommendations to combat the salamander plague in Alpine salamanders.

## Methods

We studied 40 populations between 2017 and 2022, including four populations of *S*. *lanzai* (90 individuals), 32 populations of *S*. *a*. *atra* (567 individuals), two of *S*. *atra aurorae* (28 individuals) and one of *S*. *a*. *pasubiensis* (30 individuals; Fig 1 and S1 Table). We selected sampling localities opportunistically by including those which were previously well-known alpine salamander populations or were part of previous and ongoing surveillance projects. We additionally compiled available literature data from 13 *Bsal*-screenings that included Alpine salamander populations. Opportunistic visual encounter surveys during night and days with suitable weather conditions (i.e. rain) were conducted between May and October. Besides Alpine salamanders, our sampling included syntopic caudates susceptible to *Bsal* (Alpine newt, *Ichthyosaura alpestris*; European fire salamander, *Salamandra salamandra*; in total 103 individuals, S1 Table). We excluded anurans from our sampling as they rarely carry *Bsal* in the wild [7]. During sampling, we handled individuals with nitrile gloves and changed them between individuals. Further, we physically examined each specimen for skin damages as described for *Bsal* infections in members of the genus *Salamandra* [7, 28]. Except for specimens sampled in Austria and the German site Mittenwald, we rinsed all individuals with a sterile NaCl solution (9g/l; Fresenius Kabi®) before swabbing to reduce potential inhibitors during DNA extraction. Per specimen, two skin swabs (except Austria, here it was one per individual) were taken for verification. That means, in case of a potential *Bsal*-positive result (see below for details), it was possible to validate the sample by an independent facility to avoid false positives [cf. 29]. All applicable national guidelines for the care and use of animals were followed. Handling of live specimens was granted under several protocols (Regione del Veneto, Giunta regionale, Italy: 0247416; Ministero della transizione ecologica, Italy: 0055632. 05-05-2022; ISPRA, Italy: 0016482/ AAL/Rif. Int. 13633–16162; Vorarlberg, Austria: BHBL-II960-18/2017-11, BHBR-I-7100.00-6/2017-5, II-6201-3/2017/4, BHFK-II6101-4/2017-4; Tirol, Austria: U-NSCH-11/48/15-2018, NA-16-2020, NSCH/B-367/5-2020; Schwaben, Bavaria: 55.1-8622-4/49/3; Oberbayern, Bavaria: ROB-55.1-8646.NAT_02-5-31-3; Baden-Württemberg: 55-7/8852.15-3/Uni Trier).

In samples from Austria, DNA was extracted using the ExtractMe DNA Swab & Semen Kit (Swift Analytical) following the manufacturer’s instructions. Presence of *Bsal* was tested using a modification of the screening assay described by [30] on a BioRad QX200 droplet digital PCR cycler. Primers and a probe targeting the 5.8S rRNA gene of *Bsal* were run in the FAM channel and internal control primers and a probe targeting a portion of the mitochondrial Cytochrome b gene were run in the HEX channel. The threshold for detection was set to three positive droplets. The samples of *S*. *lanzai* from Italy and France in year 2018 were extracted after [31] and processed on a BioRad CFX96 Real Time PCR Detection System following [30]. In samples from Germany and Italy (year 2022), DNA was extracted using the DNeasy Blood and Tissue kit (QIAGEN) with the following deviations from the manufacturers kit. We include a bead-beating step of 45 sec with 0,035–0,04g of silica zirconium beads (0,5mm diameter) after the addition of ATL buffer prior to enzymatic lysis. Enzymatic lysis was performed for two hours. Extracted DNA was eluted in 70μl of AE buffer. We subsequently amplified a fragment of the internal transcribed spacer region [30] in duplicate via quantitative PCR on a StepOnePlus (ThermoFisher Scientific) following the protocols of [29, 32]. We set the limit of detection (LOD) to 100 DNA copies [14]. Samples that yielded a positive signal below the LOD were verified via end-point PCR using an additional primer pair amplifying a fragment of the 28S rRNA region following the protocol of [33] on a Biometra TAdvanced (Analytik Jena) in duplicate. To avoid pseudo-replication per population, we visited all sites only once and we released all specimens at their exact capture sites after finishing sample collection. In all sampling sites, we thoroughly disinfected equipment and boots before and after entering a locality, following commonly applied biosecurity protocols [14]. We estimated prevalence following [34] under the assumption of a pathogen prevalence of 10% [35]. Further, to validate our results as well for those sites with a sample size below 30 individuals tested, we used the Bayesian hierarchical model with the same assumptions described in [36] for the entire dataset (S1 and S2 Tables). For sample sites with multiple sample years, we included only the data of the latest sample year. We calculated posterior means and 95% highest posterior densities (HPD) for our dataset. The calculated values give information on posterior probabilities of *Bsal* presence for each site. Further, they precise with 95% confidence the true value of possible *Bsal*-sites in our dataset [36]. We used R v.4.3.2 [37] for the described prevalence and Bayesian hierarchical model estimation.

For a risk estimation of *Bsal* invasion within the geographic ranges of Alpine salamanders, we built a correlative Species Distribution Model (SDM) with Maxent 3.4.1 [38, 39] in the manner described by [8] with the following modifications. We added new records from the pathogen’s invasive range adopted from [10] and used the CHELSA TraCE21k climate data [40]. For final modelling, we used an approach employing linear, quadratic and product feature classes with the bioclimatic predictors Bio2, Bio4, Bio7, Bio9, Bio10 [cf. 41]. We resampled the selected bioclimatic variables from 1x1km to 100x100m to increase the resolution for the elevational gradient using binominal interpolation in ArcGIS Pro [42, 43]. For SDM mapping, ArcGIS Pro and ArcMap (ESRI) were used. With this, we constructed a binary presence/absence distribution map of *Bsal*. For this purpose, we examined various thresholds (S3 Table) and chose three commonly used: the minimum training presence cloglog threshold (MTP, 0.0114), defining the lowest predicted suitability value for an occurrence point falling within the area of the binary model; the maximum training sensitivity plus specifity Cloglog threshold (MTS, 0,0237) which maximises the correct classification of positive and pseudo absence points and the 10^th^ percentile training presence Cloglog threshold (10^th^TP, 0,478) as a more conservative measure (by excluding outliers below 10%) [44–46]. All Maxent values above these three thresholds suggest suitability for *Bsal*.

## Results

Our molecular analysis from skin swabs revealed the absence of *Bsal* in all 758 specimens examined throughout this study. Hence, we increase the *Bsal* sampling dataset within the Alpine salamanders’ ranges to 1,137 (S1 Table). No *Bsal*-typic macroscopic skin damages were observed throughout our surveys. For several localities, sample size was too small (< 30 individuals) to draw robust conclusions that *Bsal* occurs with a prevalence of 10% (S1 Table) [23, 35]. The hierarchical Bayesian model, however, shows that up to 7,1% of sampling sites could be positive for *Bsal* in the worst case (i.e. HPD for lowest sensitivity of diagnostic test, Table 1 and S1 and S2 Figs). A single sample of *S*. *lanzai* yielded a positive signal below the LOD, which could be further rejected via non-amplification of a second primer pair. All three thresholds of the SDM suggest that the entire geographic space encompassed by Alpine salamanders is suitable to *Bsal* (Fig 1).

**Table 1:** Estimation of posterior means and 95% HPD intervals for the proportion of *Bsal*-positive sites at different *Bsal* detection sensitivities.

Sensitivity	Mean	HPD (lower and upper)
0.5	0.024	8.6e-07–0.071
0.6	0.023	1.9e-6–0.069
0.7	0.022	1.6e-6–0.067
0.8	0.022	8.5e-6–0.067
0.9	0.022	2.0e-6–0.065
1	0.021	1.7e-8–0.063

## Discussion

### Absence of *Bsal* and infection risk

Our findings suggest that Alpine salamander populations in the Alps are free from *Bsal* and go in line with earlier studies in the Alps inside (S1 Table) as well as outside the *S*. *atra* or *S*. *lanzai* ranges [23]. However, it is difficult to preclude overlooked *Bsal* outbreaks in the Alpine region with our sampling (Table 1) [36]. To stress this, for *S*. *a*. cf. *prenjensis* in Slovenia, latest sampling dates to 2015–2019. Moreover, in the Dinarides, also inhabited by *S*. *a*. *prenjensis*, the latest available sampling was in 2013 in Bosnia, showing a present and perilous knowledge gap for *Bsal* data in this region [17]. Given the recent discovery of the pathogen in Allgovia, southern Germany [20], Alpine salamander habitats are best classified as being in the “pre-invasion phase” defined by [15]. That is, prevention of pathogen introduction and spread is of high priority making urgent action needed. Moreover, *Bsal* suitability, as shown by three thresholds of our SDM, underlines our call for pre-invasion measures as it overlaps with our sampling sites, the distribution of Alpine salamanders and other syntopic *Bsal* hosts (Fig 1). However, our predictions are slightly different to those from [47] which show solely suitability along the edges of the alpine region but not in the centre. This may be due to methodological differences as we used an extended dataset of *Bsal*-records and a finer resolution [10, 47]. Still our model likely underestimates the habitat suitability for the pathogen, as *Bsal* is continuously spreading and is not in equilibrium with the environment in its invasive range [cf. 8]. Moreover, *Bsal* shows capacities to rapidly evolve, implying shifts in its ecological limits within its invasive range [12, 48].

Above all, human activity such as the amphibian pet trade (e.g. interchange of infected individuals) on a local to global or the recreational activities on a local to regional scale (e.g. unintended transport of water or soil through equipment), are likely a major long-distance vector for the salamander plague. This was demonstrated for the closely related chytrid fungus *Batrachochytrium dendrobatidis* [49] and expected for *Bsal* [e.g. 6, 8, 50]. The Alps are among Europe’s top destinations for tourists, and hence it cannot be ruled out that during outdoor activities (such as mountaineering, hiking, mountain-biking) tourists unintentionally carry *Bsal* spores into Alpine salamander habitats. *Bsal* spores can survive in soil over prolonged periods and some spores even persist in dry conditions [7]. To stress this, in [51], tourism was defined as a serious risk for amphibian pathogen introduction into naïve regions. In this regard, we consider the locally restricted *S*. *lanzai* in the Monviso Transboundary Biosphere Region, Piemont Province of Italy (Fig 1), is of particular concern, because this area is a popular travel destination for recreational (eco-)tourism [52–54], while the local endemics *S*. *atra aurorae* and especially *S*. *a*. *pasubiensis* occur in less accessible areas. However, their localities are well known among herpetological amateurs and professionals as well as nature photographers, and due to their uniqueness and rarity their sites are still frequently visited.

Despite the suggested *Bsal* susceptibility by anecdotical reports and inferred from phylogeny [4, 15, 21], it remains untested whether Alpine salamander populations respond to the pathogen and its disease in a similar way as their relative, the European fire salamander (*S*. *salamandra*). Often accompanied by mass mortality, *Bsal*-positive populations of this species dramatically decline within weeks [6–8]. *Bsal* apparently does not evenly diffuse in the landscape. Rather, European fire salamander populations neighbouring outbreaks can stay *Bsal*-free for many years [13]. Landscape heterogeneity and physical barriers to vectors (i.e. high mountain ridges and deep valleys) may play a role [55]. Hence, for the relatively wide-spread *S*. *a*. *atra*, one may perhaps assume that a salamander plague spill-over between populations is hampered or at least slowed-down in alpine environments. Moreover, populations are often naturally isolated [e.g. 18, 56]. However, if the pathogen enters a population, a rapid population collapse is likely, as Alpine salamanders locally often occur in high densities. Due to their viviparous reproductive style, compensatory recruitment is slow, as e.g. a single female in *S*. *atra* usually produces only one or two descendants every two years [56].

### Surveillance strategies

**Fig 2:**
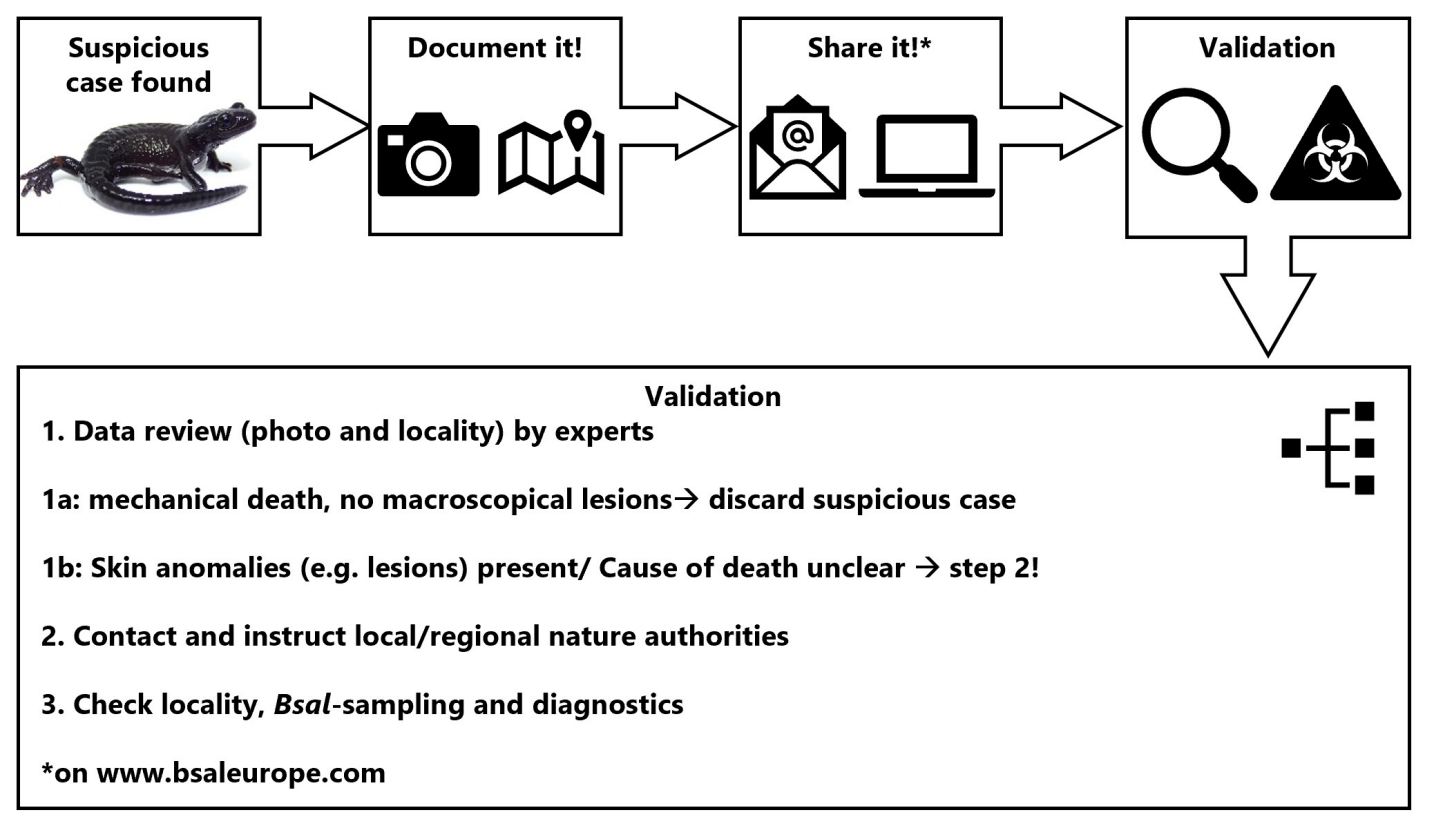
Suggested report system of suspicious cases of disease in a citizen science framework for Alpine salamanders.

Several strategies have been identified to monitor and prevent further *Bsal* spread in the Americas and Europe, while measures to successfully eradicate the pathogen once it has established are not yet available [14, 15, 56–58, F. Pasmans & A. Martel pers. comm.]. This means, that combating *Bsal* so far is only possible in the “pre-invasion phase”, which calls for urgent action in Alpine salamanders. Only some of the strategies suggested by [14, 15, 59], which we here review (Table 2), can be applied to them. The approaches proposed for other caudates (i.e. surveillance, such as swabbing of focal and syntopic amphibians, eDNA and citizen science-based approaches; prevention such as biosecurity and captive assurance colonies; population monitoring), even those in the genus *Salamandra*, are partially not applicable or are demanding in time and effort. To overcome these limitations, citizen science approaches may help as participants might be available to register sightings (Fig 2) over the entire activity period of the focal species. Therefore, it is more likely to notice Alpine salamander activity or mortality events than during temporally and spatially limited active surveillance. Citizen science has already proven effective for detecting other invasive species at an early stage [60, 61]. However, encouraging citizen science can only aid salamander conservation when the risk of human-mediated pathogen introduction is avoided by following strict biosecurity recommendations [14, 62]. Disinfection of materials could be implemented before entering and after leaving a recreational site (e.g. hiking equipment at parking areas). We encourage public *Bsal* information campaigns [cf. 63] including an App-based online reporting system for suspicious mortality events of Alpine salamanders in the wild. On a European scale, this may be implemented via online platforms commonly used across countries (e.g. BsalEurope, observation.org, iNaturalist, ornitho; Fig 2). In addition, regional or species-specific platforms may be installed. These need to be connected for rapid information exchange, which is crucial for surveillance of Emerging Infectious Diseases [64]. However, citizen science can generally only complement pathogen screening with standardized molecular tools by professionals, which should especially target syntopic, *Bsal-*tolerant hosts where pathogen presence goes unnoticed from the public. This underlines that EID surveillance and prevention generally needs stronger support by national and international decision-makers (e.g. fast-tracked permission process) to connect these different surveillance strategies in a legal framework [64].

**Table 2:** Suggested actions for the *Bsal* pre-invasion phase adapted from [14, 15, 59] for Alpine salamanders.

Strategy	Advantages	Disadvantages
**Active surveillance**		
Swabbing target species	Standard method of *Bsal*-detection; Updated overview of target populations possible	Time consuming; costly; fast analysis via qPCR required
Swabbing syntopic species	Standard method of *Bsal*-detection; Updated overview of disease status in the target habitat; may foster fast detection of EIDs because of different life history of syntopic species; fast achievement of minimum sample size of 30 individuals per population	No pathogen status on target species; Time consuming; costly; fast analysis via qPCR required
eDNA (water bodies and detritus/soil)	Non-invasive method, established for *Bsal* in water bodies; fast large scale EID detection	Water bodies: suitable only for standing water; applicable for syntopic species and adjacent habitats Soil/detritus: not yet established; Suitability unknown
**Passive surveillance**		
Suspicious cases	Including the public into conservation actions	Possible lack of interest
Raising Awareness	Includes regional and local stakeholders	
**Preventive measures**		
Captive assurance colonies (ex situ)	Buys time to develop in situ mitigation strategies against EIDs	Time and money intensive to organize and establish breeding facilities and network; identify genetic diversity of target species/subspecies at first; very low fecundity; needs a minimum number of founders
Biosecurity	reduces chances of spread of *Bsal* and other amphibian pathogens, Protocols are available	Depends on willingness of all stakeholders to implement properly; Use of chemicals may have adverse effects on humans and environment; Costs associated with communication and implementation; Cannot control for all potential routes of transmission (e.g. wildlife)
**Other**		
Population Monitoring	Detection of declines; increases detection probability of mortality events	only appropriate on a long term; time intensive

To conserve the local endemic lineages (at least *S*. *a*. *aurorae*, *S*. *a*. *pasubiensis*, *S*. *a*. *prenjensis*, *S*. *lanzai*), these actions might not be sufficient as an unnoticed introduction could lead to their rapid entire extinction [15]. Therefore, we additionally, in line with previous suggestions using ex situ strategies to reduce extirpation risk [59], we recommend evaluating the feasibility of establishing biosecure captive breeding colonies to safeguard these taxa. For some lineages (e.g. *S*. *a*. *pasubiensis*), no syntopic caudates ‐ which may act as reservoirs ‐ are known, increasing the chance for a successful reintroduction after extinction of both, the local salamander population and *Bsal*. However, little is known about captive requirements of abovementioned taxa and hence capacities need to be established early so that husbandry protocols are developed before *Bsal* might arrive.

Above all, implementing biosecurity standards in the Alpine salamanders’ range is necessary to prevent novel introductions of wildlife EIDs and their agents such as *Bsal* [14, 15].

## Conclusions

Our screening triples the existing data about non-detection of *Bsal* in Alpine salamanders and presents first information on the disease status of several endemic lineages. However, it needs to be seen as a snapshot, and can only be a first step towards a continuous survey in the future, which is urgently required. While not yet affected by the salamander plague, the SDM shows high habitat suitability over the entire range of Alpine salamanders for *Bsal*. Conclusively, the modelled suitability shows the importance for rapid preparation in these *Bsal*-naïve regions. We therefore recommend (in line with [65, 66]) to build a strong and solid cross-country network to ensure a transparent interchange, and to jointly establish an agreement how to effectively respond once suspicious cases are detected. Besides, such a network also fosters additional risk assessments, such as applied by [67–70], which need to be adapted for the alpine region. Moreover, consideration of susceptibility to pathogens that cause EIDs, like *Bsal*, in conservation assessments (e.g., red lists) is essential to prioritize conservation action.

## Supporting information

S1 Table Overview of study sites. Data is listed per country (AT = Austria, BA = Bosnia, CH = Switzerland, DE = Germany, IT = Italy, SLO = Slovenia), taxa, year of sampling as well as Prevalence per site with corresponding Credible Intervals (CI), number of samples (N: all amphibians studied per site; Numbers in brackets: sample subset of syntopic species, see Methods), Number of Bsal positives (N positive) and reference. N.A.: not assessed.
(XLSX)

S2 Table Input file for the hierarchical Bayesian model after [36]. (CSV)

S3 Table SDM results produced with MAXENT. (XLSX)

S1 Fig Estimated posterior distributions for mean prevalence of positive *Bsal*-sites within the Alpine salamander dataset. Facets refer to the sensitivity of the diagnostic test.
(TIF)

S2 Fig Estimated posterior probability of *Bsal* presence for each site. Facets refer to the sensitivity of the diagnostic test.
(TIF)
