# Supplementary figures and images for: Alpine salamanders at risk? The current status of an emerging fungal pathogen
Source: PLoS One. 2024 May 17;19(5):e0298591. doi: 10.1371/journal.pone.0298591 (PMC11101120; doi:10.1371/journal.pone.0298591)

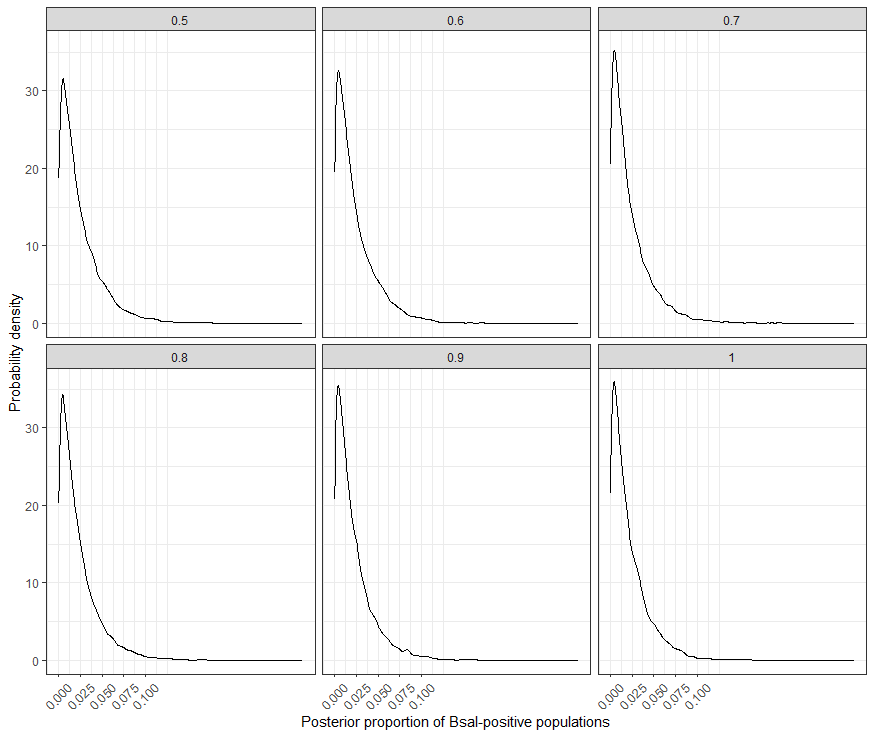

Supplement: S1 Fig — Facets refer to the sensitivity of the diagnostic test. (TIF) [file pone.0298591.s004.tif]

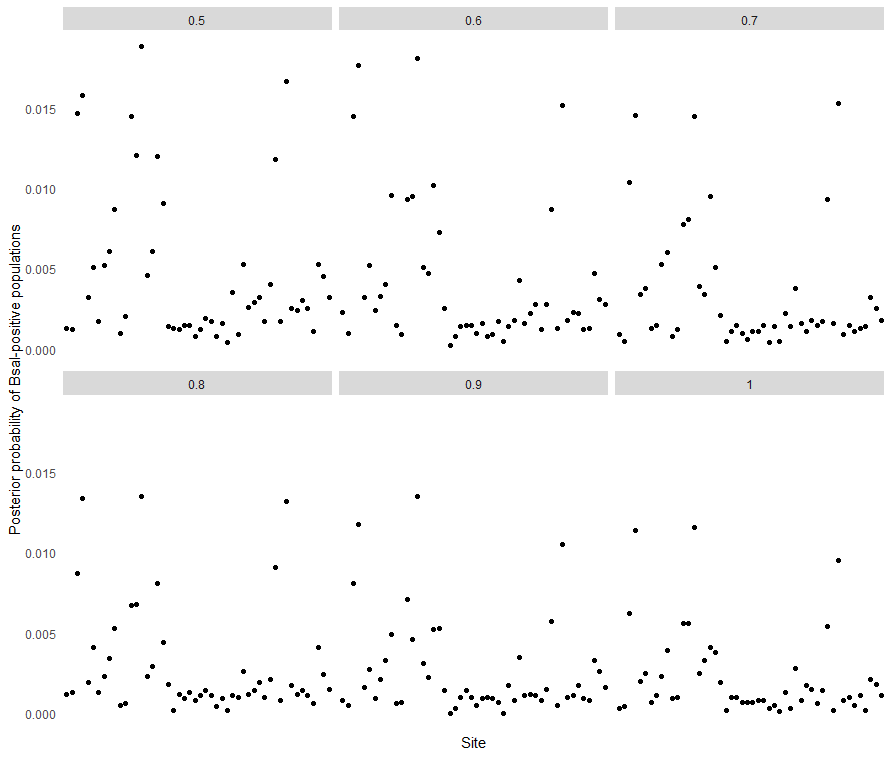

Supplement: S2 Fig — Facets refer to the sensitivity of the diagnostic test. (TIF) [file pone.0298591.s005.tif]
